# Data-Based Modeling and Control of a Single Link Soft Robotic Arm

**DOI:** 10.3390/biomimetics10050294

**Published:** 2025-05-06

**Authors:** David Abraham Morales-Enríquez, Jaime Guzmán-López, Raúl Alejandro Aguilar-Ramírez, Jorge Luis Lorenzo-Martínez, Daniel Sapién-Garza, Ricardo Cortez, Norma Lozada-Castillo, Alberto Luviano-Juárez

**Affiliations:** 1 Instituto Politécnico Nacional UPIITA, Av Instituto Politécnico Nacional 2580, La Laguna Ticomán, Gustavo A. Madero, Ciudad de México 07340, Mexico; dmoralese@ipn.mx (D.A.M.-E.); raguilarr1700@alumno.ipn.mx (R.A.A.-R.); jlorenzom1400@alumno.ipn.mx (J.L.L.-M.); dsapieng1700@alumno.ipn.mx (D.S.-G.); aluvianoj@ipn.mx (A.L.-J.); 2 Instituto Politécnico Nacional CIDETEC, Av. Juan de Dios Bátiz S/N, Nueva Industrial Vallejo, Ciudad de México 07700, Mexico; jguzmanl1500@alumno.ipn.mx; 3 Instituto Politécnico Nacional UPIIAP, C. 11 Sur 12122, San Francisco Mayorazgo, Puebla 72480, Mexico; rcortezv@ipn.mx

**Keywords:** soft robot, kinematic modeling, data based control, neural network

## Abstract

In this work, the position control of a cable-driven soft robot is proposed through the approximation of its kinematic model. This approximation is derived from artificial learning rules via neural networks and experimentally observed data. To improve the learning process, a combination of active sampling and Model Agnostic Meta Learning is carried out to improve the data based model to be used in the control stage through the inverse velocity kinematics derived from the data based modeling along with a self differentiation procedure to come up with the pseudo inverse of the robot Jacobian. The proposal is verified in a designed and constructed cable-driven soft robot with three actuators and position measurement through a vision system with three-dimensional motion. Some preliminary assessments (tension and repeatability) were performed to validate the robot movement generation, and, finally, a 3D reference trajectory was tracked using the proposed approach, achieving competitive tracking errors.

## 1. Introduction

The development of robotic systems has been highly studied in recent years to solve several difficulties that are not only related to production lines where the conditions are controlled, but also several robotic systems are focused on the solution from tasks like exploration, manipulation of fragile objects and non-conventional motion where the environment conditions are not controlled [1,2].

From these tasks, one of the most required is the exploration of constrained spaces like pipes, between walls, rubble mounts, and similar places. Classical robots are not suitable for performing the task since it is required that the robot modifies its shape to be capable of performing the exploration [3]. In this sense, compliant robotic systems [4,5,6] have been developed as an alternative to come up with robotic systems with higher adaptability via their flexibility, which can be helpful in some special applications such as human–robot interaction [7]. Robots with soft bodies (soft robots) and robots that combine soft and rigid materials can be found among compliant robots.

Concerning Soft Robots (SR), this class of robots usually replaces the rigid mechanical structures and the use of servomotors with pneumatic actuators [8], hydraulic actuators [9], shape memory alloys [10,11], magnetic actuators [12], electro-chemical actuators [13] or the implementation from cable-driven transmission of the motion from traditional actuators [14]. The main idea of the SR is that this class of actuators can perform complex movements with a single actuator that would require several actuators and a complex mechanical structure to be implemented with traditional actuators [15]. The placement or implementation of this actuator on the SR allows for a decrease in the size for specialized applications [16], implementation on wearable devices [17], and generation of complex shapes that are not feasible to be implemented with traditional actuator structures [18].

One of the main problems related to the SR is the model and control from these kind of devices, since the principles used for the actuation and the developed structures imply the presence of nonlinear dynamics, existence of hysteresis effects, delay effects by thermal or chemical processes and the presence of external perturbations [19,20]. To solve this problem, several options have been developed like the implementation of Fuzzy strategies for hysteresis compensation [21], use of the Prandtl–Ishlinskii model for compensation on a P controller [22], sliding mode controller with PID for vibration damping [23], model free iterative learning control [24], adaptive robust controllers [25], active disturbance rejection [26], Neural Network (NN) control [27], model predictive control [19], machine learning methods [20] and Data Base Modeling (DBM) [28]. This last one consists of the acquisition of a large amount of experimental data that is used for Neural Network training that does not consider the model from the system once the Neural Network is trained; this one is used to calculate actuator positions that allow the system to reach a configuration from the SR.

The outline of this work is as follows: Section 2 describes the process of fabrication from the SR and describes the main idea behind their actuation system using two agonist-antagonist systems. Section 3 describes the problems related to the model and control from the SR associated with the inverse kinematics. Section 4 includes the DBM developed on this work that considers hysteresis compensation. Section 5 describes the experimental setup to implement the DBM in the SR and consists of repeatability and tension tests to validate the quality of the SR. Section 6 describes the results related to the instrumentation required for the DBM, the GUI used to control the SR, and the evaluation from the DBM for a tracking task. Section 7 discusses the conclusions related to the feasibility of the DBM on SR applications.

## 2. Problem Statement

The main problem is formulated as follows: Design and implement a single soft arm robotic system with three-dimensional positioning to be controlled via kinematic data-based control in trajectory tracking tasks, with validation through a vision-based motion capture system.

## 3. Soft Robot Testbed

**Figure 1:**
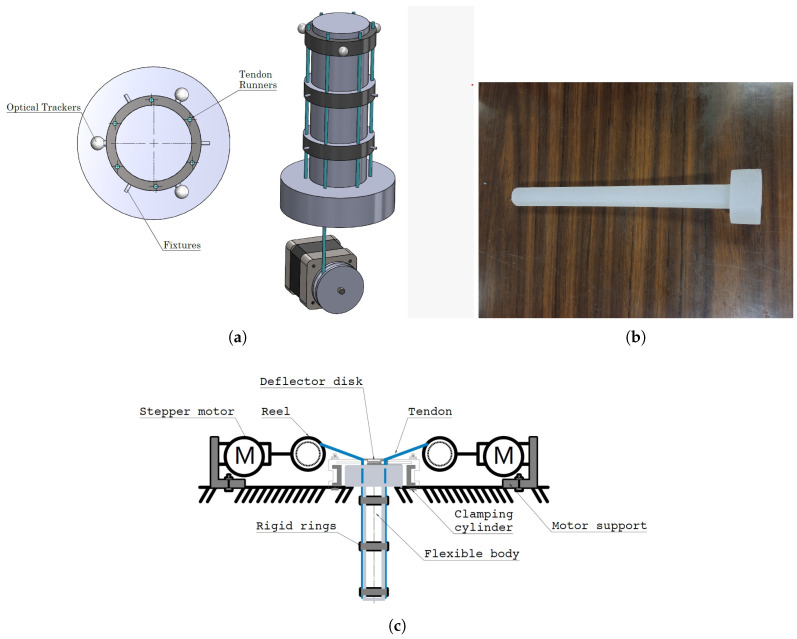
Testbed schematic proposal. (**a**) Graphic representation of soft robot. (**b**) Soft robot. (**c**) Diagram of the actuation system.

The present proposal considers an experimental platform for a cable-driven flexible robot manipulated by four cables controlled by two to four stepper motors (see Figure 1a). In addition, the following features are given:

- Link robot material with a modulus of elasticity less than 10 MPa, ensuring a low-rigidity material with a nonlinear effect concerning position is sought, where a geometry that behaves the same way in any direction of movement will be sought.
- Two or more stepper motors (actuators) to generate a sequence of position objectives to provide trajectory tracking.
- Provide trajectory tracking with absolute error less than 50 mm.
- Minimize wear caused by the sampling process. That is, shorten the experimental process to avoid damaging the robot’s material that could alter its original physical dimensions and kinematic relationship.

Based on the above considerations, each platform part is analyzed as follows. First, the soft body is cylindrical (see Figure 1b) and made of $Eco-Flex^{TM}$ 00−30 material with the following characteristics (see Table 1):

On the other hand, six 0.6 mm nylon cables (tendon cables) are incorporated into the soft body from its base and along the body through three rings. These rings have the objectives of (1) holding the tendon cables, (2) adjusting the movement space of the soft body, and (3) holding some tracking markers. The actuation system is also based on the tension change (agonist–antagonist mechanism) on two to four tendon cables carried out using four stepper motors, one motor for each tendon (see Figure 1c). Then, a structure (box) aims to hold the soft body at one of its ends, the four motors, the connection and control diagram, and the power supply.

Finally, to support the acquisition of soft body position information, three markers are added to the non-fixed end ring of the soft body, and four markers are added to the structure (box) to verify the measurements of the soft body markers.

Four Rantec brand Nema17 stepper motors carry out the tension on four of the six tendon cables (see Figure 1c).

## 4. Modeling and Control

The approach to control using data-based methods benefits enormously from the research stream on neural networks or deep learning. The development of new techniques combined with the production and adoption of parallel processing has allowed us to produce solutions for problems that are classically difficult to model, such as the modeling and control of soft robots, which are naturally infinite-dimensional systems [29].

To model the kinematics of the soft robot, an artificial learning paradigm is adopted, which assumes the existence of a pattern that guides the distribution of observations of a phenomenon (denoted as the objective function). Based on the data, the determination of the approximation hypothesis corresponds to three fundamental elements: **representation, evaluation, and optimization** [30].

The representation corresponds to the hypotheses from which the resulting function will be obtained. A set of parameters can represent each of these hypotheses, so navigating the set of hypotheses can be reduced to gradually modifying these parameters. Here, the representation is provided through neural networks as universal approximators, even with nonlinearities such as hysteresis, which is typical in soft robotic systems. However, the effectiveness of the learning process is closely related to the designed tests to obtain a sufficiently rich set of data that can effectively discriminate the kinematics with a set of data as reduced as possible.

The evaluation of the approximation scheme is proposed through the Mean Square Error index, or MSE, denoted as

(1) $$\begin{array}{cc} MSE(q) & =\frac{1}{N}\sum_{n=1}^{N}{\left(P\left(q_{n}\right)-p_{n}\right)}^{2} \end{array}$$

where $q_{n}$ is the actuation parameter vector, $p(q_{i})$ is the model prediction, $p_{n}$ the the actual position, and *n* the number of tests.

Optimization is the procedure of using the collected information in the evaluation to update the hypothesis parameters and improve its behavior. Among the deep neural network training algorithms, the Stochastic Gradient Descent (SGD) and the Adaptive Moments (or Adam) make up an effective and popular combination alternative through their fast convergence rate and robustness against learning architecture variations [31,32]. The SGD sets the parameter update as a fractional multiple of the average error gradient for the set of samples. That is:

(2) $$\begin{array}{c} \theta ⟵\theta -\frac{\lambda }{m}\nabla_{\theta }E\left(f\left(x;\theta \right),y\right) \end{array}$$

where θ is the set of parameters, λ the learning rate, *E* is the loss function, *f* is the model, (x,y) are the spread sample pairs, and *m* is the amount of spread samples. Adam modifies the SGD algorithm that induces extra terms in the gradient to improve the parameter update while compensating for the initialization bias. The following update expressions are derived from this procedure:

$$\begin{array}{cc} \begin{array}{c} g_{\theta } \end{array} & \begin{array}{c} \leftarrow \frac{1}{m}\nabla_{\theta }E\left(f\left(x,\theta \right),y\right) \end{array}\\ \begin{array}{c} s \end{array} & \begin{array}{c} \leftarrow \rho_{1}s+\left(1-\rho_{1}\right)g_{\theta } \end{array}\\ \begin{array}{c} r \end{array} & \begin{array}{c} \leftarrow \rho_{2}r+\left(1-\rho_{2}\right)g_{\theta }\cdot g_{\theta } \end{array}\\ \begin{array}{c} \hat{s} \end{array} & \begin{array}{c} \leftarrow \frac{r}{1-\rho_{1}^{t}} \end{array}\\ \begin{array}{c} \hat{r} \end{array} & \begin{array}{c} \leftarrow \frac{r}{1-\rho_{2}^{t}} \end{array}\\ \begin{array}{c} \theta \end{array} & \begin{array}{c} \leftarrow \theta -\frac{\lambda \hat{s}}{\sqrt{\hat{r}}+\delta } \end{array} \end{array}$$

where $g_{\theta }$ is the modified gradient according to the samples number, and *s* and *r* are estimations of the first and second moment, respectively. $\tilde{s}$, $\tilde{r}$ are the corrected estimators, $\rho_{1},\;\rho_{2}$ are the moment decay parameters (with values 0.9 and 0.999, respectively), *t* is the number iteration and the stabilization constant is δ ( $10^{-8}$) to avoid zero division.

**Automatic differentiation**

Auto differentiation is a process that helps us obtain the time derivative of each output with respect to the input, forming the transformation Jacobian necessary for the kinematic model construction. This facilitates the implementation of gradient-based optimization methods for having optimal parameters via the derivative of the error with respect to these parameters. This mechanism allows the construction of the Jacobian matrix of the neural model. Thus, the Jacobian of the network evaluated at a point approximates the Jacobian of the objective function around the same point.

**Active sampling for the network training**

The effectiveness of the data-based modeling and control is related to the dataset’s quality. To avoid some drawbacks due to repeatability errors or low quality sampling processes, active sampling was implemented which consists of organizing an ensemble of models and training them on the initial data set such that when there are regions not covered by the initial data set, a list of candidate vectors is produced in the search space, and the predictions of each model in the ensemble are generated for each of these candidate vectors to improve the network prediction in these regions.

**Inverse kinematics through the Jacobian pseudo-inverse**

By definition, the Jacobian of a manipulator relates the velocity of the actuation parameters to the velocity of the end effector as follows:

$$\begin{array}{cc} \dot{p} & =J\left(q\right)\dot{q} \end{array}$$

where *q* is the actuation parameters vector, J(q) is the Jacobian matrix and *p* is the end effector position. The inverse problem requires knowledge of the Jacobian inverse, which may not be of square dimensions. In the last case, the Moore–Penrose pseudo-inverse can be used to get the time derivative of *q*, or approximately via an iterative procedure, an increment of the actuation values, denoted as Δq. This increment allows us to design a control law based on the desired trajectory to design a control action to generate the actual end effector trajectory to track the desired one.

### 4.1. Data Set Assembling

Two arrays of the *Q* and *P* sequences are created from the SR marker position data, representing the motor actuation and the marker position values, respectively. It is important to consider that the SR operation samples are slower than the acquisition (vision) system samples for the present work. So, subsampling is performed through a linear interpolation of the *P* array samples to generate a pairing with the *Q* array data. Mathematically, we have:

$$\begin{array}{c} \begin{array}{c} p_{j}=p_{i}+\frac{t_{j}}{T_{P}}\left(p_{i+1}-p_{i}\right) \end{array} \end{array}$$

### 4.2. Algorithms for Modeling Control

The training of the data-driven model will be carried out through two main approaches: (*a*) supervised learning neural networks, (*b*) algorithms to minimize the samples needed to achieve the objective. The above was developed mainly in the following points:

**1**. The model architecture and the parameter space in which each point corresponds to a different model are defined. [(*a*)]
**2**. Selection of a set of admissible initial parameters. [(*b*)]
**3**. A model is found that approximates the distribution obtained in an initial sampling of the robot kinematics. [(*a*)]
**4**. Once the initial information is obtained, the samples are adjusted to improve the model. [(*b*)]
**5**. Updating the robot’s current samples to continue improving the model. [(*b*)]

More specifically, for step **1**, a multilayer perceptron-type differentiable NN was considered using the hyperbolic activation function with three hidden layers.

In step **2**, the *Model Agnostic Meta Learning* (MAML) [33] method is used to obtain a set of initial parameters (see Algorithm 1). In the procedure described in Algorithm 1, line 11 is the most computationally complex since it requires storing the complete graph of operations from when the original parameters are adapted until the gradient is evaluated. To simplify this step in the meta-adjustment, a first-order approximation called Reptile [34] is used (see Algorithm 2).

For step **3**, based on a set of sinusoidal test trajectories for the SR, a model is generated via a back propagation Neural Network using the descent gradient rule (see Algorithm 3).

In step **4**, active sampling is performed based on the initial sampling, where the most effective samples are selected to improve the model. This procedure defines a set of independently initialized models to identify new samples that produce larger variance values within the predictions set (see Algorithm 4).

For step **5**, behavioral data is collected to refine the model further. This is done using Algorithm 3.

Finally, the inverse kinematics method takes the requested positions as inputs and returns the corresponding actuation vector. From steps **1**–**5**, a differentiable approximation of the robot’s forward kinematics is obtained, so it is possible to obtain the Jacobian of the model (Algorithm 5), and further using an iterative method for the inverse kinematics (Algorithm 6).

**Algorithm 1:** Meta-Adjustment of the set of initial parameters

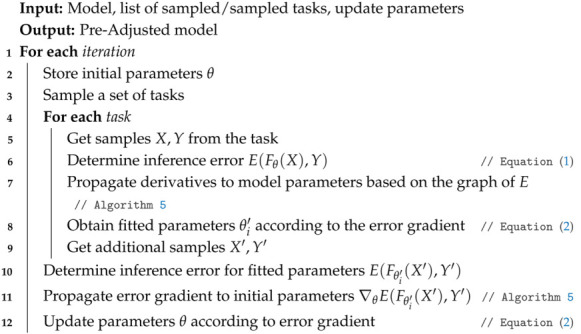



**Algorithm 2:** Meta-Adjustment: Reptile method

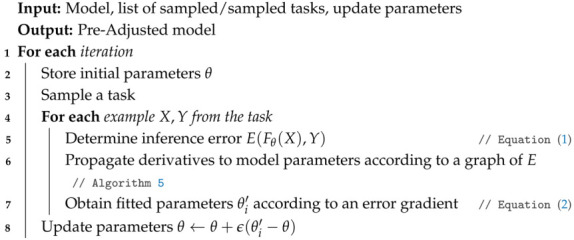



**Algorithm 3:** Individual Model Training

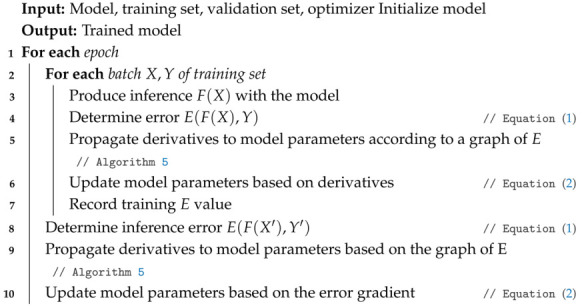



**Algorithm 4:** Active Sampling

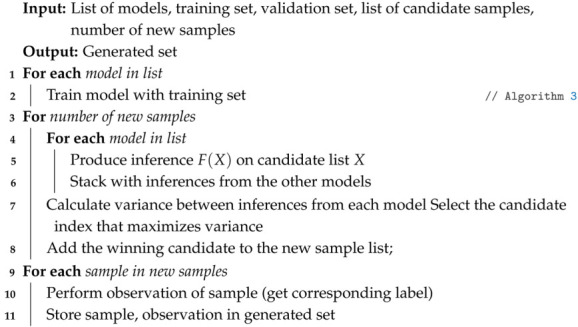



**Algorithm 5:** Obtaining derivatives of a function by self-differentiation (reverse)

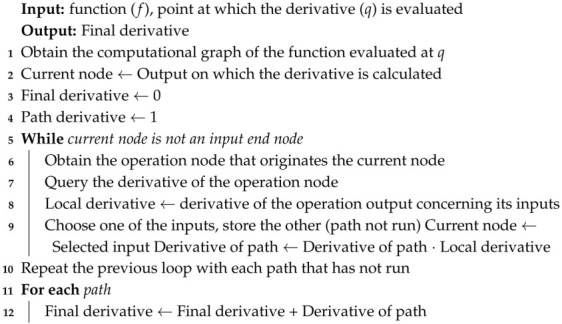



**Algorithm 6:** Iterative inverse kinematics by pseudo-inverse of the Jacobian

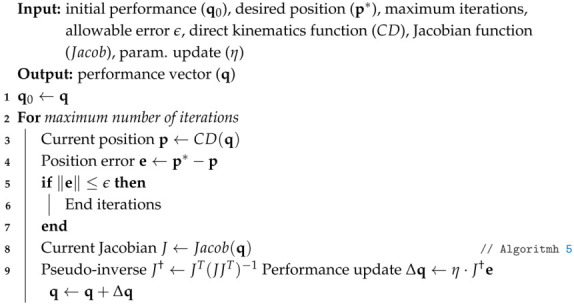



## 5. Experimental Setup

A graphic user interface (GUI) was developed on Python to implement the data acquisition, analysis, and control from the SR. This interface allows the user to perform the steps required to perform the DBM technique. The software provides the implementation of the NN using PyTorch V1.13 to define the model and the propagation method. The inverse kinematics were obtained using the self-difference method.

**Figure 2:**
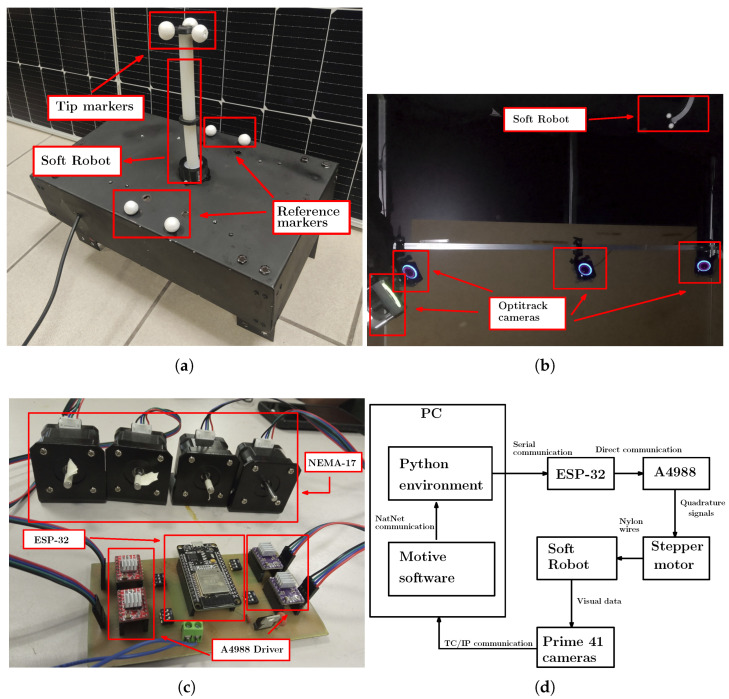
Experimental setup from the SR for the implementation of the DBM. A specific description is listed as: (**a**) Markers for the video tracking used to position data acquisition. (**b**) Markers for the video tracking used to position data acquisition. (**c**) Power stage used for the control of the NEMA-17 step-motors. (**d**) Data communication used for the instrumentation of the SR.

Implementing the data-based modeling technique for the designed SR requires considerable data from the tip position in the space of the SR for training the NN. To solve this problem, three markers placed on the tip of the SR are used to define a rigid body with a centroid, and four markers on the base of the robot are used to determine the reference coordinate system, as shown in Figure 2a. These markers are identified using an acquisition system based on 8 Optitrack Prime 41 cameras placed around the SR, as shown in Figure 2b. The position of the markers in space is computed via the software Motive to be used on the DBM. The data is obtained on Motive software, so its transmission to the environment that contains the NN is performed with the implementation of a data streaming TCP-IP via the NatNet package to provide the data to the Python software V3.10.

**Figure 3:**
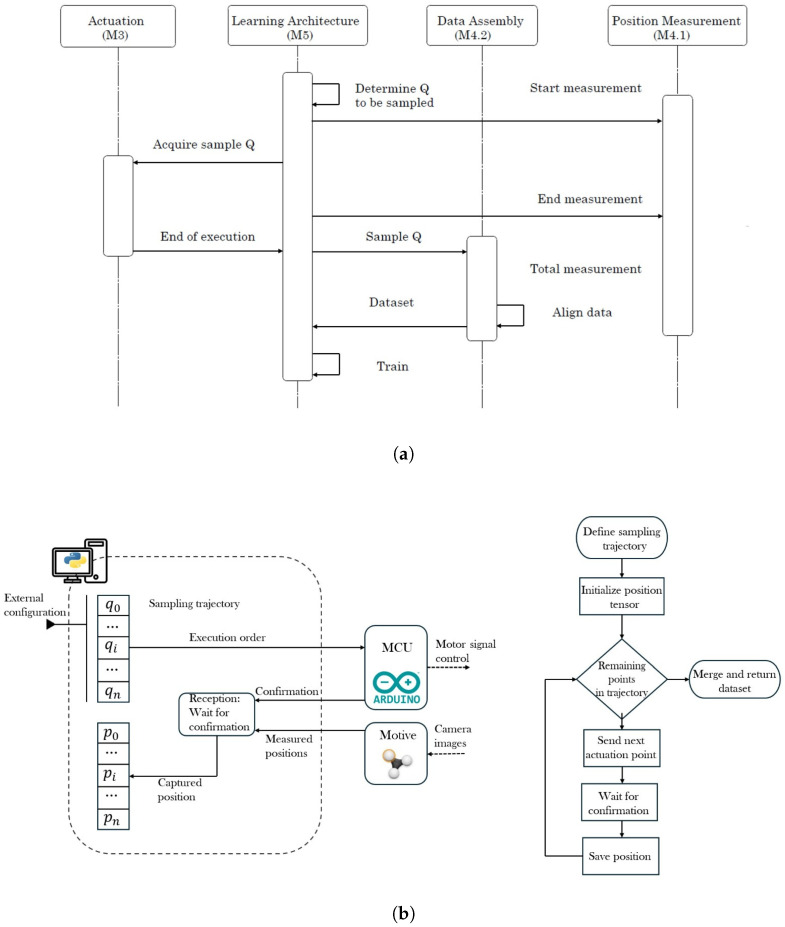
(**a**) Sample capture diagram. (**b**) Data assembly diagram.

Since the movement from the SR is performed using a double agonist-antagonist mechanical system that is generated from step-motors NEMA-17, which posses steps accuracy to $5^{{}^{\circ}}$ and are transmitted to the robot via nylon cables with a thickness from 0.6 mm. The workspace defined for the tip from the SR consists of 7 cm over the X axis, 3 cm over the Y axis and 7 cm over the Z axis. A4988 Drivers control the step-motors for each motor; all the drivers are controlled by a single ESP-32 board programmed with the ESP-IDF software, and the connections are shown in Figure 2c. The data information is sent from the computer to the ESP-32 via a serial communication protocol to activate the step-motors and perform the motion. The protocol is configured in a single direction since the DBM is implemented. Some schematic diagrams of the sample capture procedure and the data assembly are provided in Figure 3.

The configuration of the data communication required for the DBM from the SR is shown in Figure 2d. It can be noticed that there exists a feedback loop between the different stages to verify the tracking process once the DBM is performed.

### 5.1. Repeatability Test

**Figure 4:**
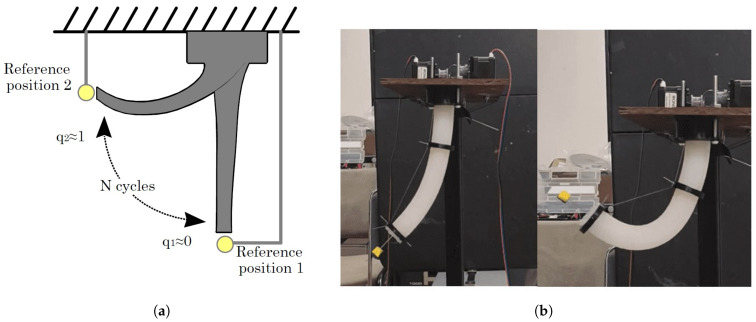
(**a**) Repeatability test schematic. (**b**) Actual experimental test arrangement.

A repetitive motion analysis is performed to verify the soft robot’s behavior under prolonged use. To do this, the centroid of a marker placed at the free end of the soft robot is observed, and a 2D movement is performed from one point $q_{1}$ to another $q_{2}$; see Figure 4.

**Figure 5:**
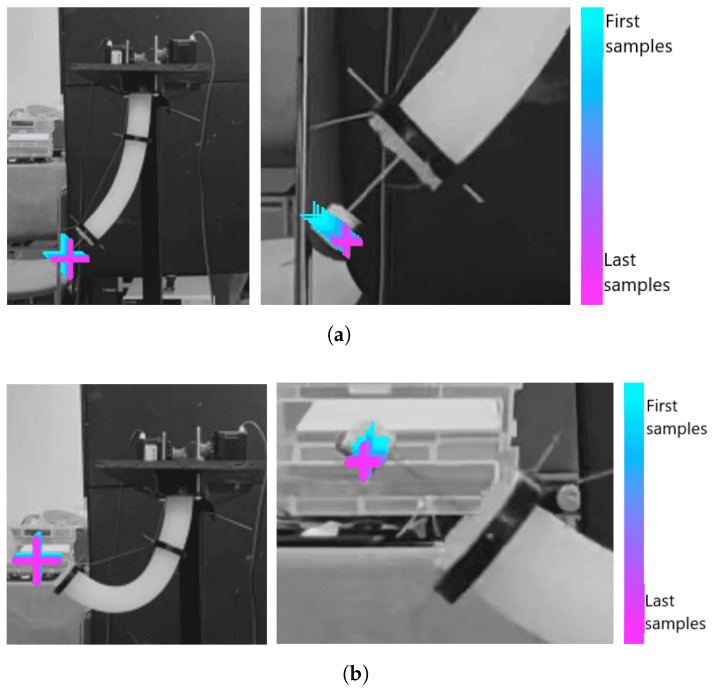
Repeatability test. (**a**,**b**) show two different test positions for the experiment. The left side of each figure shows the complete movement while the right side describes the position dispersion of $q_{2}$ marker in a zoom.

With the above, two experiments are carried out, one qualitative and another quantitative. The first one, without measuring exact positions but visually and qualitatively, the variation in the position of the marker centroid, the dispersion of the marker position after 100 cycles of movement is observed, as depicted in Figure 5.

According to qualitative observations, the measurement process relative to the cable needs to be recalibrated after 100 repetitions. This ensures the operation without the necessity of an external vision system.

The second experiment seeks to analyze the repeatability, fatigue, and deformation capacity of the soft body under the action of the cables. Considering the configuration of Figure 4a,b, 100 movements were repeated, starting at rest, then a contraction, and finally, rest. In Table 2, the most considerable variation is in the returns to rest, where the difference between the minimum and maximum recorded values has a standard of 3.4 mm, below 5 mm.

From the above, it is concluded that the SR has adequate performance up to 100 cycles, after which there may be a loss of rigidity in the soft body, or a stretching of the cable with which it acts.

### 5.2. Tension Test

**Figure 6:**
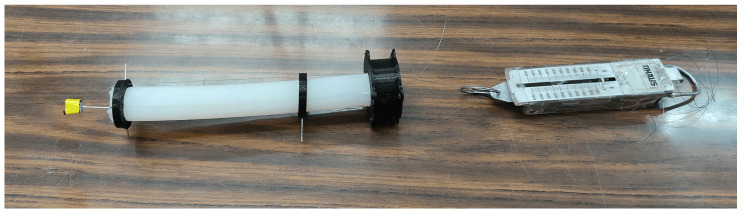
Tension test.

A test determined the minimum force required for the SR to move to a desired position (see Figure 6).

**Figure 7:**
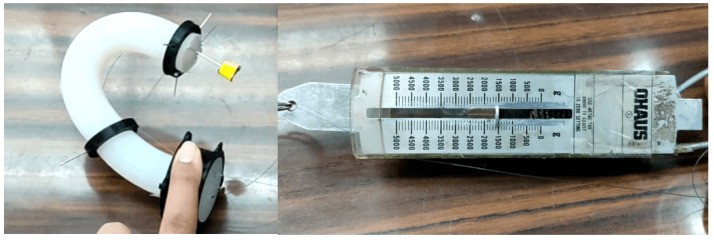
Soft Robotic system and dynamo-meter.

The behavior of the SR depends on the quantity and location of the rings; for the case study, three rings (reels) were considered, where one is fixed at the base, and the other two are located at the tip and 15 cm from the latter see Figure 7.

After different evaluations, it was determined that 1.5 kgf is required for the SR to move to its maximum tension, which is important in the actuator selection features.

### 5.3. Data Collection Test

This test aims to analyze information regarding the reliability of the data obtained via the vision system. To do this, two elements are analyzed: measurement reliability and data alignment. In both cases, the fixed markers of the box (reference markers) are considered with known information about their position and distance between them. Additionally, the Euclidean norm is used.

For the first analysis, the vision system obtains the frame-by-frame position measurements of each marker. Thus, an average measurement of these captures was obtained for 30 different frames (see Table 3). Subsequently, the physical distance between the markers is compared with the average data from the vision system (3). The above is summarized in Table 4.

**Figure 8:**
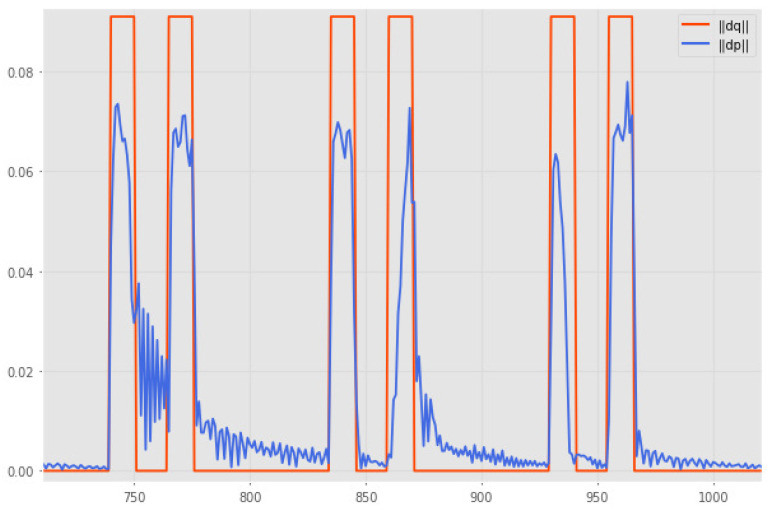
Actuation-position data alignment test.

On the other hand, the second analysis seeks to verify the data alignment error of less than 100 ms, approximately equivalent to a sample. For this, a trajectory of repeated contractions of rest periods is generated. Then, the temporal difference between the norms of the instantaneous differences for each vector is analyzed for the data set obtained. A portion of this test can be observed in Figure 8, where it is observed that the variations in both curves remain mostly simultaneous, with occasional separations that do not exceed one sample frame as desired.

## 6. Results

**Figure 9:**
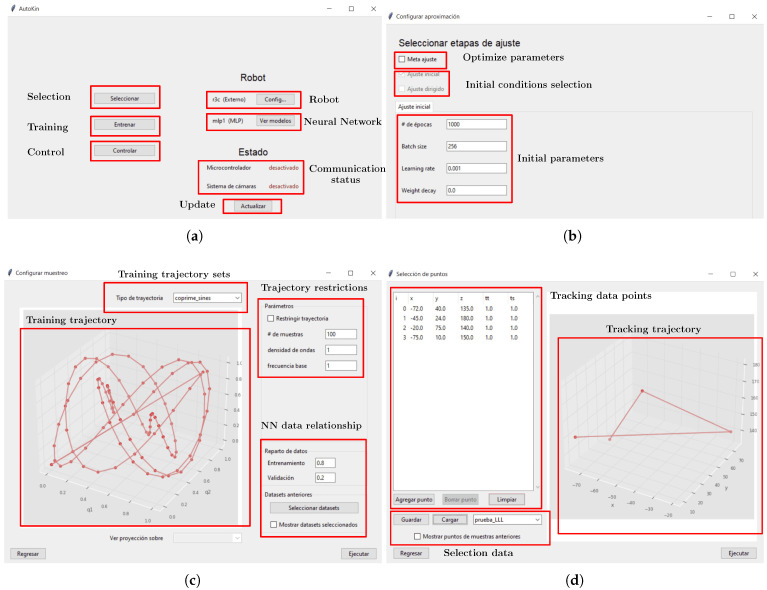
Graphical User Interface for the DBM. A specific description is listed as: (**a**) Main screen from the GUI divided on several sections. (**b**) Selection for training parameters from the NN. (**c**) Experimental data acquisition and training of the NN. (**d**) Selection of data points for the tracking task.

The implementation of the GUI that is used to implement the DBM has a main screen shown in Figure 9a, which allows the selection between the different stages. The section **Selection** allows the user to choose between previously trained robots or create a new robot; at the same time, the model for the robot could be selected on the section **Robot** and the NN model to be trained can be modified on section **Neural Network**. The parameters from the NN could be optimized with the implementation of a meta-heuristic algorithm, as is shown in Figure 9b, with a selection of initial parameters or using a directed adjustment based on previously trained robots. The section **Training** allows the user to implement the training process from the NN via the acquisition of experimental data, as shown in Figure 9c. On this, there could be a selection between a data set of test trajectories that could be acquired via the SR and shown on a graphical representation. These trajectories could be restricted based on the particular properties from the SR. Additionally, the relationship between the number of data used for training and validation is set. The **Control** allows us to define a set of points that the robot must follow, and based on the NN execute interpolate, the articular positions of the step-motors required to perform a physical routine from the SR such their tip reach all the positions proposed, as is shown in Figure 9d which has a list of the data points, a graphical representation of the trajectory on the space and a menu to save or load different trajectories. The **Communication status** section identifies if communication exists between the Motive software and the ESP-32 device to perform the required tasks; if the communication is unavailable, the training and control process cannot be performed. The section **Update** allows the software to retry the connection with Motive and the ESP-32 to perform the DBM.

To verify the quality of the NN with hysteresis compensation training, a set of points on a complex trajectory is proposed to be tracked by the SR. At the same time, two cases of the same NN are evaluated: The first one corresponds to the NN without training parameters optimized using a meta-heuristic algorithm. The second one corresponds to an NN with training parameters optimized via the meta-heuristic algorithms. To evaluate the quality of the tracking, the Medium Error (ME) index is defined as follows:

$$\begin{array}{c} \begin{array}{c} \mathrm{ME}=\sum_{i=1}^{n}\sqrt{{\left(x_{i}^{*}-x_{i}\right)}^{2}+{\left(y_{i}^{*}-y_{i}\right)}^{2}+{\left(z_{i}^{*}-z_{i}\right)}^{2}} \end{array} \end{array}$$

where *i* is the number of data point to be evaluated, *n* is the total number of data points to be tracked, $x_{i}^{*}$, $y_{i}^{*}$ and $z_{i}^{*}$ corresponds to the desired positions to be tracked for the data point *i*. At the same time, $x_{i}$, $y_{i}$, and $z_{i}$ are the measured positions for the SR tip estimated using the Motive software for the data point *i*.

**Figure 10:**
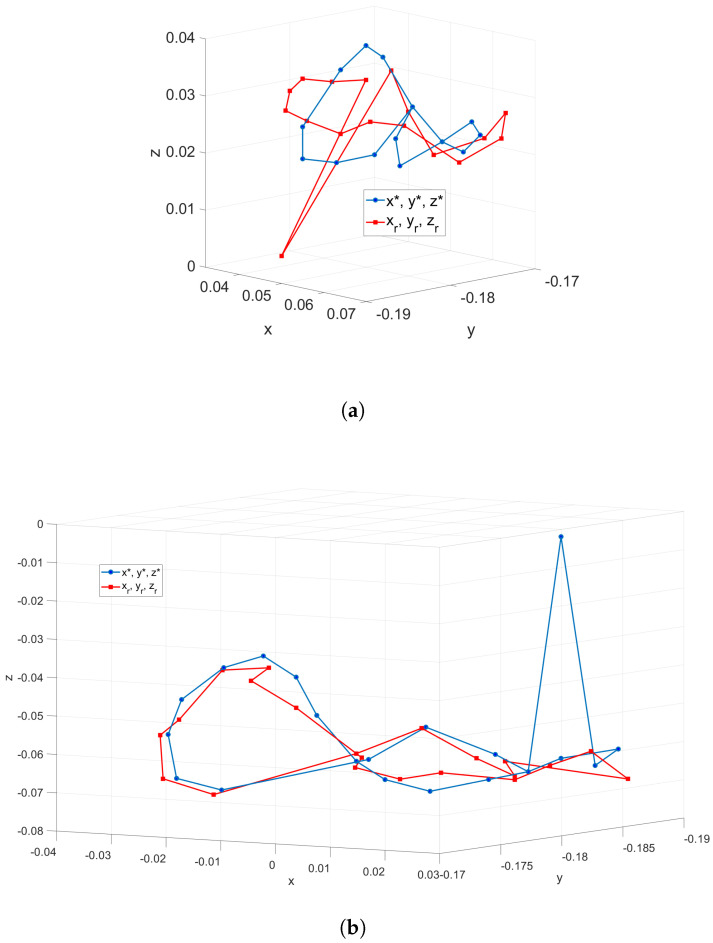
Tracking task of the SR robot based on the DBM. A specific description is listed as: (**a**) Tracking task using a NN without training parameter optimization. (**b**) Tracking task using a NN with training parameter optimization.

The tracking task for the first case is shown in Figure 10a, which allows us to notice the existence of several points that the SR does not well reach. The ME has a value from 8.61 mm that is related to the inadequate training of the NN in the implementation from the DBM. The second case is shown in Figure 10b and has an ME value of 4.37 mm, which allows for the verification of an improvement in the quality of the trained NN and the feasibility of the DBM for the control of SR. The tracking process is performed without feedback on the control since the vision system is just applied to validate the quality of the tracking. The trained DBM could be used for the SR under conditions that do not possess visual feedback if previous training and validation is performed. On the other hand, the numerical data of the trajectory tracking error for both cases are shown in Table 5 and Table 6.

From Table 5 (test without training parameter optimization), notice that the mean square errors for the axes *x*, *y*, and *z* are 0.000247, 0.000039, and 0.00354644, respectively. The improvement due to the addition of the training parameter optimization can be noticed in Table 6 whose mean square errors for the axes *x*, *y*, and *z* are reduced to 0.000047, $1.47778\times 10^{-05}$, $9.07222\times 10^{-05}$. This test motivates using the complementary optimization process in the data-based modeling procedure.

## 7. Conclusions and Future Work

The design and control of soft robotic systems is still an active research topic whose complexity demands new alternatives of modeling and control in which data-based modeling or evolutionary computation approaches can lead to competitive results in trajectory tracking tasks, as shown in the presented manuscript. Vision systems are a valuable method of pose measuring, but in practice, they can be complicated to implement due to effects such as occlusion, which can demand expensive measuring systems. In this sense, alternative sensing techniques such as the innovative scheme reported in [35] can be helpful. Exploring sensor fusion approaches for this class of robots is a challenging topic for robotic systems with soft actuators and soft robots in general [36]. With a simultaneous application of robust control approaches, these approaches may improve the general performance of these infinite-dimensional systems.

It can be observed that the proposal improves the approximation of the model using NN with the use of the Reptile algorithm as training parameter optimization for a better search, reducing the tracking errors from 62% (*y* axis tracking error) to 97% (*z*-axis tracking error), respectively.

As presented, the data-based analysis does not involve loads in the robot, nor disturbances. Therefore, addressing this aspect is considered as future work in the context of Data-Driven Model Predictive Control as a first attempt, for which the reported analysis can ensure robust stability in the case of linear systems (see [37]). The challenge here is to adapt the scheme for a class of soft robots that may demand additional steps to develop a suitable model with dynamic control that can incorporate other robust aspects and approaches in the modeling, estimation, and control.

## Figures and Tables

**Table 1:** Soft arm specification.

Parameter	Value
Length	220.00 mm
Small diameter	18 mm
Big diameter	40 mm
Young Module	0.6 MPA
Viscosity	20,000 cps
Shore hardness	30 A

**Table 2:** Repeatability verification test results.

	Standard Deviation (x,y,z) [mm]	Difference Between Minimum and Maximum Measure (x,y,z) [mm]
Rest	(0.9, 0.4, 0.4)	(2.7, 1.1, 1.8)
Contraction 1	(0.1, 0.1, 0.7)	(0.6, 0.5, 1.9)
Contraction 2	(0.3, 0.1, 0.1)	(1.3, 0.5, 0.7)
Contraction 3	(0.2, 0.2, 0.2)	(0.8, 0.7, 0.8)

**Table 3:** Markers location on the box.

	Location		
**Markers**	**X (m)**	**Y (m)**	**Z (m)**
Marker 1	0.161041188	−0.020186031	−0.159941094
Marker 2	−0.008419781	−0.014420938	−0.314508125
Marker 3	−0.147030469	−0.028594563	0.127964938
Marker 4	−0.309365563	−0.025150563	−0.033201844

**Table 4:** Verification location makers results.

	Marker 1 to Marker 2	Marker 1 to Marker 3	Marker 3 to Marker 4	Marker 2 to Marker 4
Distance (m)	0.223898108	0.415348481	0.229835392	0.412088233
Actual (m)	0.225	0.415	0.23	0.413
Error (m)	0.001101892	0.000348481	0.000164608	0.000911767

**Table 5:** Tracking error results of Figure 10a (case without training parameter optimization).

Step	$e_{x}$	$e_{y}$	$e_{z}$
1	0	−0.001	−0.002
2	0.003	0.001	0.001
3	−0.001	−0.002	0
4	−0.002	−0.002	0
5	0.008	−0.001	0.0588
6	−0.006	0	0.003
7	0.005	0	0.001
8	0.002	0	0.002
9	−0.002	−0.002	0.001
10	0.002	−0.003	−0.003
11	0.001	−0.002	−0.004
12	0.001	−0.002	−0.004
13	−0.001	0	−0.001
14	0.006	0.001	−0.001
15	0.006	−0.001	0.001
16	−0.001	0	0.003
17	−0.002	−0.001	0
18	−0.004	−0.002	0.004

**Table 6:** Tracking error results of Figure 10a (case with training parameter optimization).

Step	$e_{x}$	$e_{y}$	$e_{z}$
1	0.011	0.01	0.025
2	0.007	−0.003	−0.01
3	0.004	−0.003	−0.008
4	0	0.001	0.002
5	−0.001	−0.001	0.003
6	0	−0.003	−0.003
7	−0.003	−0.003	−0.002
8	−0.004	0	0.003
9	0	0.001	0.003
10	0.003	−0.001	−0.005
11	0.003	−0.002	−0.004
12	0.005	−0.003	−0.005
13	0.008	−0.002	−0.001
14	0.01	0.001	0.005
15	0.011	0.002	0.007
16	0.008	0.002	0.005
17	0.011	0	−0.003
18	0.011	0.01	0.025

## Data Availability

The raw data supporting the conclusions of this article will be made available by the authors on request.

## Abbreviations

The following abbreviations are used in this manuscript:

SR	Soft Robot
DBM	Data Based Modeling
NN	Neural Network
GUI	Graphic User Interface
PID	Proportional Integral Derivative
MSE	Mean Square Error
SGD	Stochastic Gradient Descendent
Adam	Adaptive Moments
MAML	Model Agnostic Meta Learning
